# Liver Transplantation as a Cornerstone Treatment for Acute-On-Chronic Liver Failure

**DOI:** 10.3389/ti.2022.10108

**Published:** 2022-03-17

**Authors:** Martin S. Schulz, Wenyi Gu, Andreas A. Schnitzbauer, Jonel Trebicka

**Affiliations:** ^1^ Department of Internal Medicine I, Goethe University, Frankfurt, Germany; ^2^ Department of General and Visceral Surgery, University Hospital, Goethe University, Frankfurt, Germany; ^3^ European Foundation for Study of Chronic Liver Failure (EF-Clif), Barcelona, Spain

**Keywords:** liver transplantation, decompensated cirrhosis, liver cirrhosis, ACLF, acute-on-chronic liver failure

## Abstract

Acute-on-chronic liver failure (ACLF) is a distinct clinical syndrome, characterized by acute decompensation (AD) of liver cirrhosis, severe systemic inflammation, intra- and extrahepatic organ failures, and a high short-term mortality. Liver transplantation (LT) is a potentially life-saving treatment for patients with decompensated liver cirrhosis and, due to the high mortality rates, particularly for ACLF patients. In the last decade, a plethora of studies has produced compelling evidence in favor of LT in ACLF, demonstrating high post-LT survival rates and excessive waitlist mortality. The importance of LT in these patients is underscored by the fact that no specific therapy for ACLF is available yet, rendering expeditious life-saving LT to be the only feasible treatment option for some ACLF patients. This review aims to provide an overview on pathophysiology, clinical trajectory, and clinical management of ACLF and to delineate the current literature regarding perspectives and limitations of LT as a life-saving treatment option for ACLF patients.

## Introduction

**FIGURE 1:**
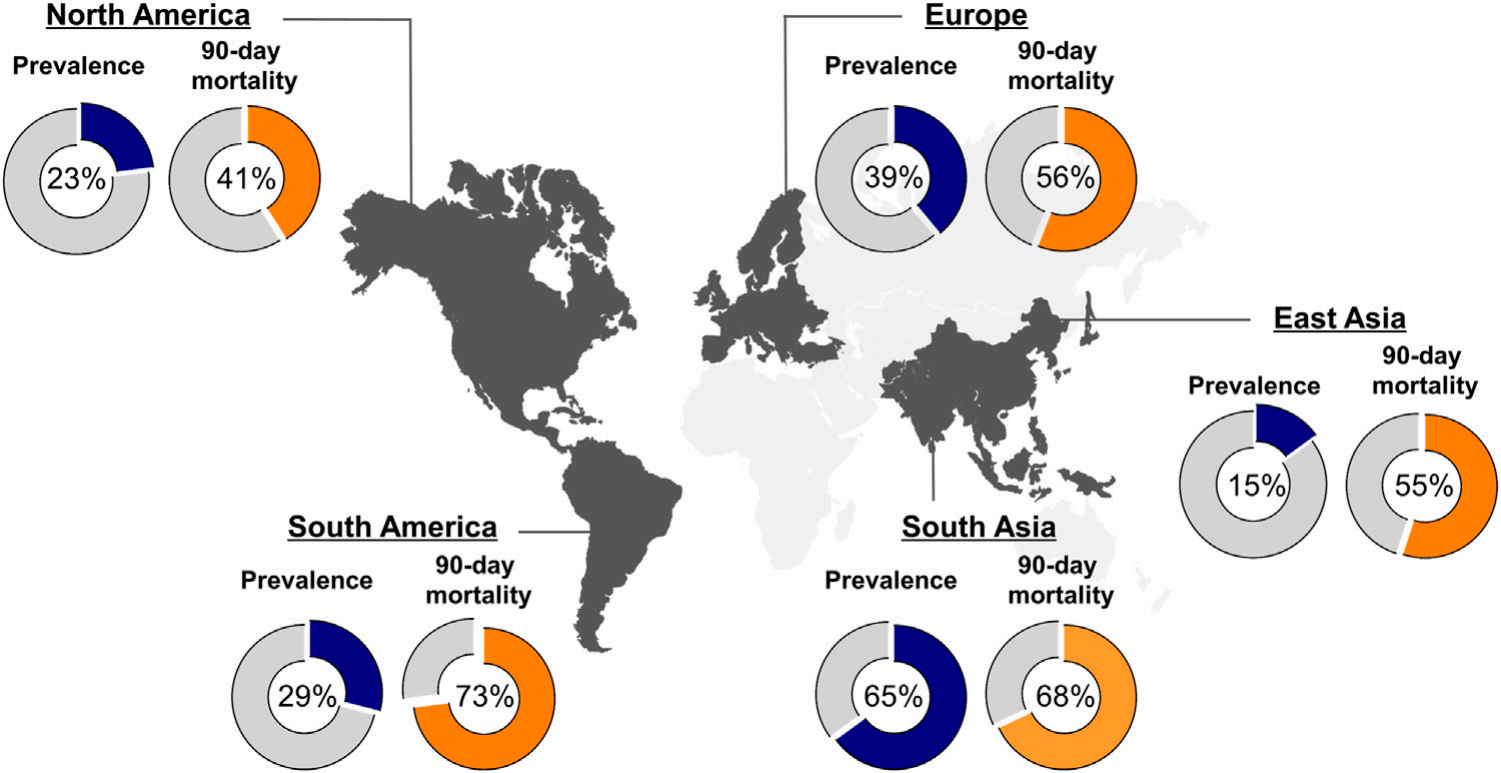
Global prevalence and 90-day mortality of ACLF. Figure displays the global prevalence of ACLF (blue piechart), according to EASL-CLIF criteria, and 90-day mortality rates (orange piechart) depending on the geographical region as reported by Mezzano et al. (11).

Liver cirrhosis constitutes a significant public health burden worldwide. It is associated with a high morbidity and a significant loss of disability-adjusted life-years (1–3). Acute decompensation (AD), defined by the onset of cirrhosis-related complications and hospitalization, is a watershed moment in a patient’s clinical course and is associated with a marked decline in survival (4). Recent studies have suggested that AD defines a heterogeneous syndrome with distinct clinical phenotypes and not a unidimensional continuum, ending in ACLF (5–7). While clinical trajectories significantly differ between these phenotypes, a considerable fraction of patients with AD progress to pre-ACLF or present manifest acute-on-chronic liver failure (ACLF). Severe systemic inflammation (SI) is the hallmark of ACLF, a crucial driver in disease progression (8, 9). ACLF is defined by acutely decompensated cirrhosis with development of extra- and/or intrahepatic organ failures and it is associated with a median transplantation-free 28-day mortality of 32.8% (10). Moreover, a recent study demonstrated ACLF to be highly prevalent worldwide in patients admitted to the hospital with AD (see Figure 1) (11, 12). Although patients with defined ACLF undergo liver transplantation (LT), to date, presence of ACLF and ACLF severity are not specifically prioritized in organ allocation.

Due to scarcity of donor organs, strong competition exists for patients on the waiting list for liver grafts, and patients with decompensated cirrhosis must also contest with patients listed for other indications with time-sensitive match MELD score, especially with hepatocellular carcinoma (HCC). MELD score-based allocation systems were designed to stratify waiting list patients with decompensated cirrhosis and to allocate liver grafts following the ‘sickest first’ principle. However, in recent years, limitations of these allocation systems have become apparent, particularly because current prognostic models do not adequately take into account that prognosis and dynamics differ distinctly between AD phenotypes and ACLF.

In the last decade, several studies have evaluated post-transplant outcomes in patients receiving LT with ACLF. The aim of this review is to provide an overview of our current understanding on pathophysiology, clinical trajectory, treatment options and prognosis of ACLF and to review recent literature on LT as a life-saving treatment option in patients with ACLF.

## Definitions of AD and ACLF

AD is defined by the onset of cirrhosis-related complications, such as development of ascites, gastrointestinal hemorrhage, hepatic encephalopathy or bacterial infection leading to hospitalization (6). The development of AD constitutes a decisive time point and a “prognostic watershed” in the clinical course of cirrhosis (13). The trajectory of end-stage cirrhosis is commonly shaped by these decompensating events, whereby the first episode of AD leads to a significant reduction of the median survival time from 12 to less than 2 years (4, 14). In 30% of patients, AD progresses to development of hepatic and/or extrahepatic organ failures, which, together with a severe systemic inflammatory response, are the hallmarks of ACLF (10, 15, 16). ACLF is considered a distinct clinical syndrome, highly prevalent worldwide, and it is associated with a high short-term mortality, rendering it a global public health problem (17). The Chronic Liver Failure Acute-on-Chronic Liver Failure in Cirrhosis (CANONIC) study determined major risk factors in patients with AD, which were associated with a high short-term mortality. Derived from the findings of the pioneering CANONIC study, the Chronic Liver Failure-Sequential Organ Failure Assessment (CLIF-SOFA) score and the Chronic Liver Failure Consortium Organ Failure (CLIF-C OF) score were developed (10, 18) (see Table 1). According to the EASL-CLIF definition, patients present manifest ACLF in case of:

1. Single kidney failure (serum creatinine ≥2 mg/dl)
2. Single organ failure combined with kidney dysfunction (serum creatinine ranging from 1.5 to 1.9 mg/dl) and/or mild-to-moderate HE
3. Presence of two or more organ failures

**TABLE 1:** – CLIF- Sequential Organ Failure Assessment (SOFA) score defining thresholds for organ failures (bold) to assess ACLF severity (20).

CLIF-sequential organ failure assessment (SOFA) score					
Organ failure	0	1	2	3	4
Liver (bilirubin, mg/dl)	<1.2	≥1.2-<2.0	≥2-<6.0	≥6.0-<12.0	**≥12.0**
Kidney (creatinine, mg/dl)	<1.2	≥1.2-<2.0	**≥2-<3.5**	**≥3.5-<5.0**	**>5.0 or RRT**
Cerebral (HE grade)	No HE	HE grade I	HE grade II	**HE grade III**	**HE grade IV**
Coagulation (INR or PLT count)	<1.1	≥1.21 < 1.25	≥1.25-<1.5	≥1.5-<2.5	**>2.5 or PLT count ≤20.000**
Circulatory (MAP, mmHg and vasopressors)	≥70	<70	Dopamine ≤5* or dobutamine or terlipressin	Dopamine >5* or E ≤0.1* or NE ≤0.1*	Dopamine >15* or E >0.1* or NE >0.1*
Lung					
PaO2/FiO2	>400	>300–≤400	>200–≤300	**>100–≤200**	**≤100**
SpO2/FiO2	>512	>357–≤512	>214–≤357	>89- ≤214	**≤89**

**TABLE 2:** Clinical characteristics, prognosis and therapy options for patients with SDC, UDC, pre-ACLF and ACLF, according to findings of the PREDICT study (6).

	Stable decompensated cirrhosis (SDC)	Unstable decompensated cirrhosis (UDC)	Pre-ACLF	ACLF
Systemic inflammation	Minor	Moderate	Severe	Highly severe
Complications	Benign clinical course	Primarily portal hypertension-driven complications	Incipient organ dysfunctions	Manifest (multi-)organ failure(s), sepsis, IMC/ICU
Prognosis	Recompensation, discharge	Readmission due to AD	Development of ACLF after approx. 14 days	Organ failures, intensive care
Therapy	Out-patient clinic	Management of complications, consider LT evaluation	Evaluation for LT	Rapid LT, possibly ELS as briding-to-transplant
LT within 12 months	11.8%	16.7%	15.1%	—
1-year mortality without LT	9.5%	35.6%	67.4%	—

**FIGURE 2:**
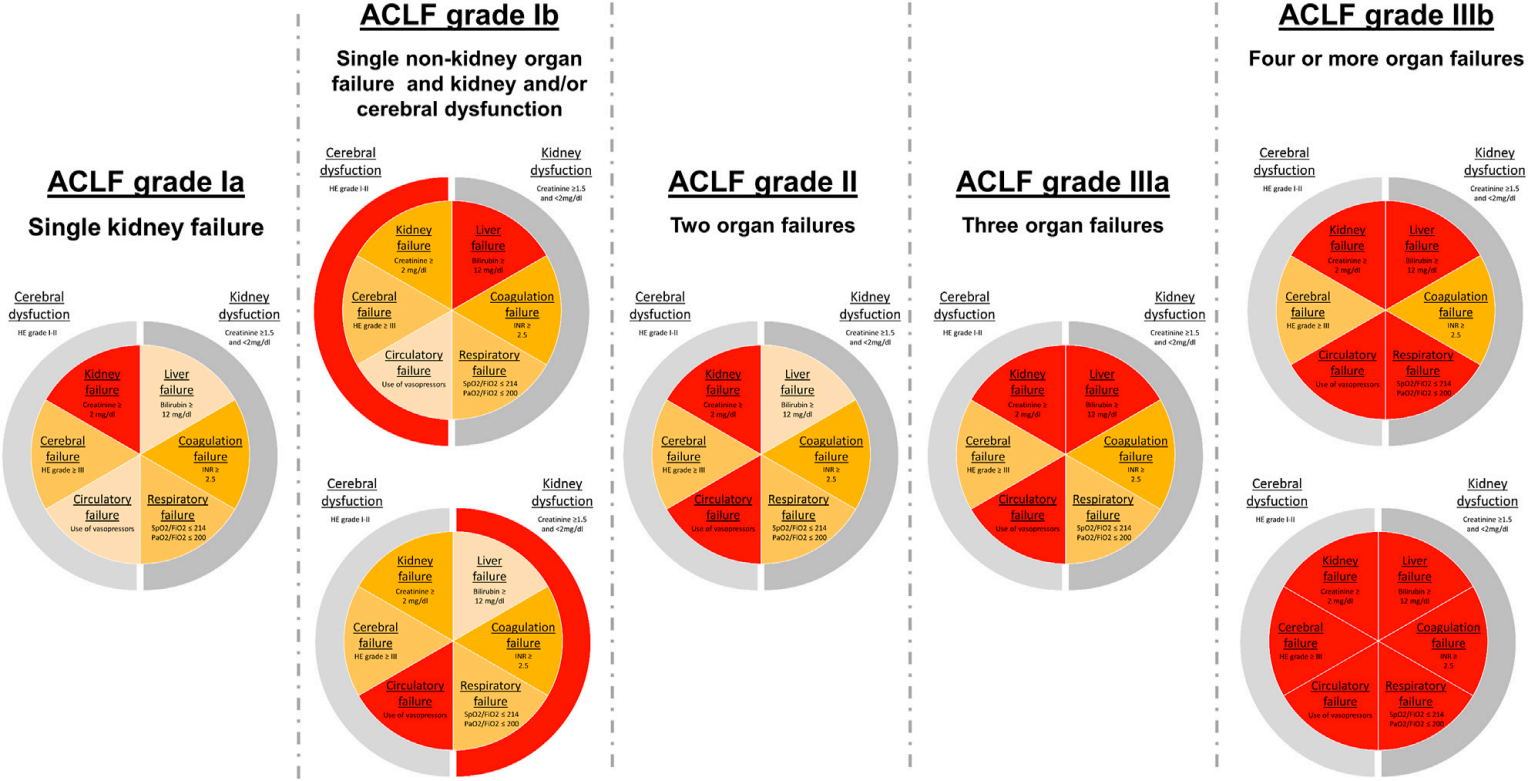
ACLF grades according to EASL-CLIF criteria. Classification of ACLF grades based on organ failure assessment by the adapted Chronic Liver Failure—Organ Failure (CLIF-OF) score (18). Pie charts show organ failure constellations in ACLF patients and the corresponding ACLF grade. Central slices display organ failures (OF), while outer slices represent relevant organ dysfunctions. Cut-off values for defined OFs, according to the CLIF-OF score, are displayed in each slice.

This definition provides a higher mortality than sepsis in cirrhosis, which clearly defines a severe clinical situation. After ACLF development, the clinical course of patients varies. While some show rapid clinical deterioration, others improve towards resolution of ACLF. Recently, a large meta-analysis of global epidemiological data found that ∼35% of patients admitted to hospital due to acutely decompensated liver cirrhosis, in fact presented defined ACLF at admission, according to EASL-CLIF criteria (11). These findings underscore the global impact of ACLF and the challenge that its clinical management poses to hepatologists and ICU physicians. Outcome is largely determined by ACLF severity, which is defined by the presence and the number of organ failures. Patients with ACLF grade 1 show a 90-day mortality of 41%, while patients with two organ failures (ACLF grade 2) or three and more (ACLF grade 3) show an even higher mortality rate of 55% and 78%, respectively (19). In contrast, 90 day-mortality in patients with AD is reported to be 14% (7). Table 1 displays thresholds for defined organ failures according to the CLIF-SOFA score, while Figure 2 shows clinical constellations of organ failures in ACLF and their respective ACLF grading (20).

Although no globally accepted homogenous definition of ACLF has been established to date, the principles of the different operating definitions according to the geographic region, the European EASL-CLIF, the North American NACSELD or the Asian Pacific APASL-AARC definition, mirror the differences in clinical practice while highlighting similar principles of organ failures, SI and high short-term mortality. In future years, the community needs to develop and homogenize a uniform definition, which will be acceptable worldwide.

### Precipitating Events of Acute Decompensation

The development of AD and ACLF is frequently caused by precipitating events, most commonly bacterial infection, alcohol-induced hepatitis, gastrointestinal bleeding or toxic encephalopathy (21). In most cases, patients show either bacterial infection and/or severe alcoholic hepatitis, alone or in combination, upon onset of decompensation. Importantly, recent data shows that the type of precipitating event does not determine patient outcome but, instead, the number of precipitants does. The PREDICT study found that ACLF patients with two or more precipitants showed a significantly higher 90-day mortality (63.4%) than patients with one (49.7%) or no determinate precipitant (42.2%) (21). In up to 60% of AD patients and 29–40% of ACLF patients, no precipitant could be identified (5, 21).

A large meta-analysis published shortly before the multicentric PREDICT study found bacterial infections to be the most prevalent precipitating event world-wide. This meta-analysis included 30 studies, analyzing data of 140,835 patients with AD and 43,206 with ACLF, defined by EASL-CLIF criteria (11). Interestingly, the authors were able to map geographical heterogeneity of ACLF prevalence and mortality rates as well as preceding trigger events. In line with the findings of PREDICT, this study also reported alcohol to be the second most frequent ACLF trigger in European study cohorts after bacterial infection, while in other geographical regions, namely East Asia and North America, alcohol consumption was the most common trigger. Viral infections played a minor role as ACLF triggers in Asia (10–12%) but were almost non-existent in other regions of the world (0–1%).

### Clinical Courses of AD and ACLF

In recent years, large prospective studies, such as CANONIC and PREDICT, have provided corroborating data on the proposed systemic inflammation hypothesis, suggesting SI to be a major driver in the progression of AD to ACLF and a crucial determinant of a patient’s clinical course (6, 10, 15, 22). The development of cirrhosis-related complications and organ failures depend on a shared pathophysiological background, which is largely determined by progression of SI (7, 9). Importantly, the grade of SI is not only associated with disease severity but also with overall patient survival (23, 24).

Recently, the PREDICT study revealed that AD constitutes a heterogeneous clinical condition with distinct clinical phenotypes (6). These clinical phenotypes are characterized by a distinct pathophysiology and are associated with a markedly different prognosis. Therefore, a novel classification has been proposed by the authors of the PREDICT study, dissecting these distinct clinical courses of AD. Patients with stable decompensated cirrhosis (SDC) represent most patients admitted with AD. These patients show detectable but low SI, present cirrhosis-associated complications less frequently, are more likely to be recompensated quickly and have a lower 1-year mortality risk (6, 25).

In contrast, patients with unstable decompensated cirrhosis (UDC) suffer primarily from portal hypertension-driven complications, show a higher risk of recurrence of AD and a significantly increased risk of death (6). Although, compared to SDC, UDC is associated with higher SI, data suggests that severe PHT is the main pathophysiological driver and the hallmark of UDC. Interestingly, UDC patients present a higher prevalence of bacterial infections, such as spontaneous bacterial peritonitis, which can in turn perpetuate decompensating events and negatively affect the further clinical course. The third clinical course of AD determined by the PREDICT study is pre-ACLF, which constitutes a distinct clinical phenotype and is characterized by development of ACLF within 90 days. These patients show rapid progression of SI compared to UDC and significantly higher short-term mortality (6). It is now well recognized that SI is a crucial driver of disease progression, possibly acting in synergy with other organ-specific pathomechanisms to mediate organ dysfunctions, ultimately facilitating the development of ACLF, which, indeed, is demonstrated by the newly described clinical entity of pre-ACLF (6, 7, 10).

### Pathomechanisms in AD and ACLF

In recent years, an emerging body of evidence has established SI as a key driver in AD and ACLF disease progression (7, 9, 26–28). While clinically significant portal hypertension (PHT) is the main driver in compensated advanced chronic liver disease (cACLD), recent studies suggested extensive activation of SI as determining further disease progression, aggravating and accelerating development of organ failures and ACLF (29). PHT-associated congestion as well as splanchnic endothelial dysfunction further aggravate gut epithelial barrier permeability (26, 30–32). Translocation of bacterial components and their metabolites is considered to cause bursts of SI by systemic exposure to gut microbiome-derived pathogen-associated molecular patterns, so-called PAMPs, presumably triggering acute decompensation events and ACLF (33–35). Indeed, emerging evidence has identified PHT-driven gut epithelial permeability as the critical driver of SI (9, 34).

**FIGURE 3:**
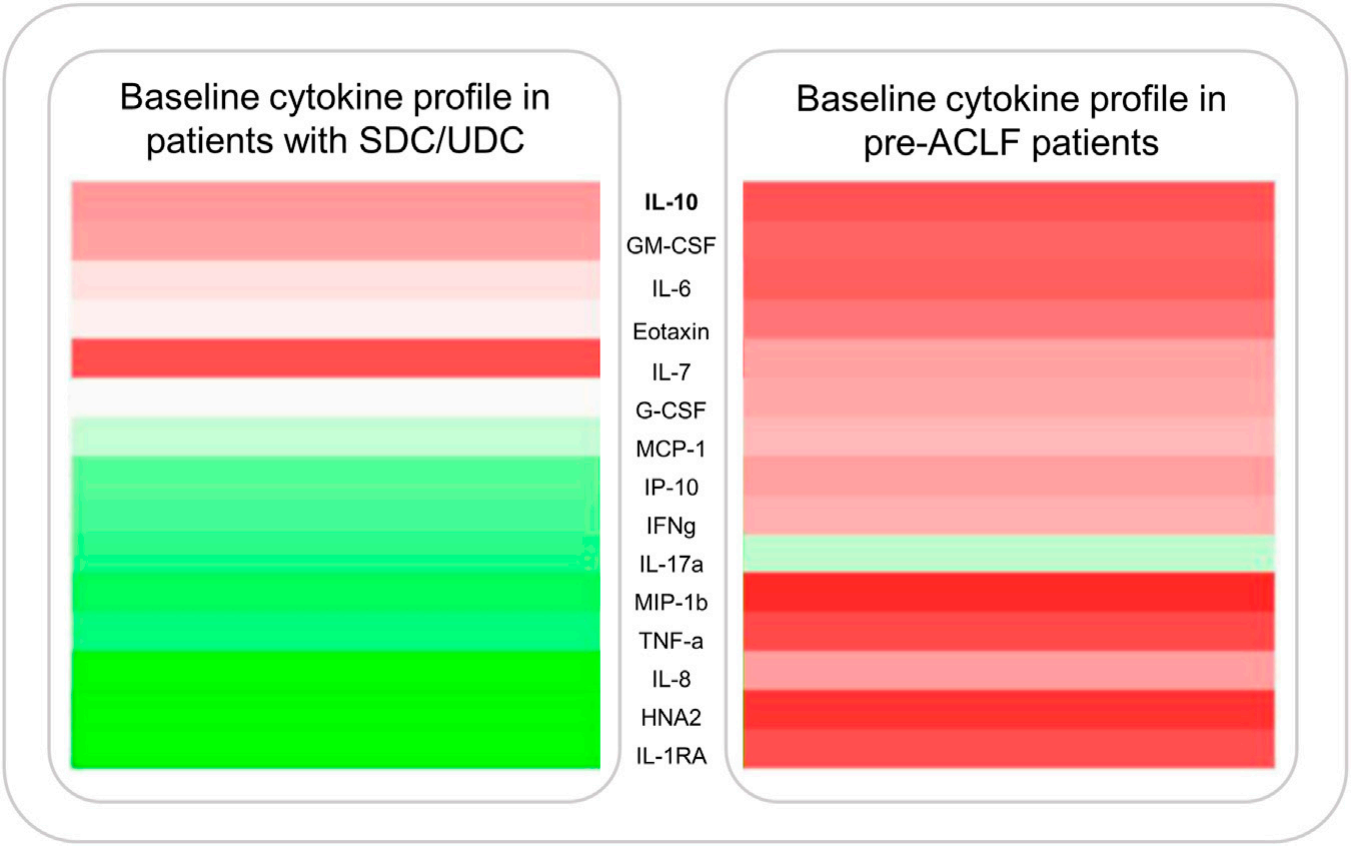
Cytokine expression profiles displayed as a heatmap in patients with AD and pre-ACLF. Figure shows median plasma levels of various pro-inflammatory cytokines at enrollment of 503 patients admitted with SDC/UDC or pre-ACLF. Data published by Trebicka et al. (27).

A recent study demonstrated progressively increasing pro-inflammatory cytokine concentrations among different AD phenotypes, being most severe in pre-ACLF (see Figure 3) (27). Patients with manifest ACLF showed high concentrations of pro-inflammatory biomarkers, such as interleukin (IL)-1ra, IL-6, IL-8, tumor necrosis factor (TNF)-α or irreversibly oxidized albumin (HNA2), which positively correlate with poor short-term survival (27). A sustained systemic inflammatory state is an energetically highly expensive process. Metabolome analysis in patients with decompensated cirrhosis has demonstrated inflammation-driven systemic catabolism, whiles manifest ACLF is characterized by severe disruptions of cell and energy metabolism and a severe catabolic state (9, 28). ACLF patients show disrupted lipid metabolism and impaired β-oxidation as well as disrupted oxidative phosphorylation and ATP synthesis (28). Thus, accumulating free fatty acids (FFAs), reactive oxygen species (ROS) and other metabotoxins presumably promote mitochondrial dysfunction, thereby accelerating metabolic disruption and cellular dysfunction. Inflammation-induced metabolic disruption and mitochondrial dysfunction, resulting in hypometabolism of peripheral organs, is presumed to complement traditional organ-specific mechanisms to perturb organ function, thereby promoting the development of organ failures and ACLF progression through these immunopathologic effects (9, 36, 37). The systemic inflammation hypothesis has severely broadened our pathophysiological understanding by complementing traditional paradigms of acute decompensation. This is paralleled by the developments in the last decade, not only regarding the changing etiologies of cirrhosis but also by a decrease in PHT-driven complications and an increase in SI-mediated decompensation (3).

### Management of ACLF Before LT

Despite the high short-term mortality of ACLF, no specific treatments are available to improve patients’ clinical course. The main principle in management of ACLF is to identify and treat the precipitating event, diagnose and treat associated complications, provide supportive therapy and, in some cases, facilitate organ support (20, 38). In patients with a determinable precipitant, early identification and adequate therapy is paramount (5). Ideally, patients with ACLF should be monitored. While monitoring is more feasible in IMC or ICU units, remote monitoring, e.g., of heart rate variability, may also be a solution in these patients as shown recently in a collaborative study (39). As the PREDICT study has demonstrated, bacterial infections and severe alcoholic hepatitis are the most frequent precipitating events in European patient populations (21). Thus, 50% of ACLF patients show infection, either as a precipitating event or as a complication, and in patients with ACLF grade, prevalence of bacterial or fungal infection increases up to 70% (5). Once bacterial infection is confirmed, early initiation of a broad empirical treatment, with consideration of local resistance spectrums, is most critical (40, 41). A globally increasing prevalence of multidrug-resistant organisms (MDROs), particularly multidrug-resistant Gram negative bacteria, poses additional challenges for clinical management (13). Notably, patients with ACLF more frequently present infections with extensive drug-resistant organisms and also show a lower infection resolution rate (42). Ideally, empirical antibiotic therapy should be adjusted to microbiological results as soon as possible.

The second most frequent precipitant in ACLF, according to the PREDICT study, is severe alcoholic hepatitis. These patients show a similar clinical course and comparable outcomes to patients with precipitant bacterial infections (21). For patients with severe alcoholic hepatitis, initiation of prednisolone therapy is often indicated. However, steroid response rates are negatively correlated to the number of organ failures at baseline (43). The Lille score can be used to identify patients who lack response to steroids early on in treatment (44). Nevertheless, in specific programs, transplantation may present an option in therapy of refractory severe ASH (45).

In cases of acute variceal hemorrhage, a new treatment option in transjugular intrahepatic portosystemic shunt (TIPS) placement has emerged, complementing the standard medical treatment of early administration of a vasoconstrictor (e.g., terlipressin or octreotide) and endoscopic therapy (20). A recent multicenter observational study identified ACLF at admission to be an independent predictor of mortality and risk of rebleeding in patients with acute variceal bleeding (46). In these patients, pre-emptive (early) TIPS placement showed a significant benefit in 42-day and 1-year survival (46). This is a clear demonstration that PHT plays a crucial role as a driver of AD.

Depending on the geographical region, viral hepatitis can constitute a rather frequent cause of ACLF, particularly in Asian countries (11). In cases of hepatitis B virus infection or reactivation, an immediate initiation with a nucleoside or nucleotide analogue is indicated.

Conventional dialysis devices are highly effective in restoring fluid homeostasis and removing toxic hydrophilic substances from the circulation. However, these devices are unable to eliminate non-hydrophilic compounds, which accumulate in the body in the context of liver failure and ACLF (47). Therefore, extracorporeal liver support systems (ECLS) were developed, which can eliminate albumin-bound compounds. ECLS can be considered as a bridging strategy, especially in patients eligible for liver transplantation, but also in selected patients as a definite treatment to improve organ function. However, more evidence in needed. Two systems, albumin dialysis (MARS®) and fractionated plasma separation and adsorption system (Prometheus®) have been evaluated in large randomized controlled trials (RCTs) among ACLF patients. In both controlled trials, data did not show a significant benefit in overall patient survival (48, 49). Notably, at the time when these initial studies were conducted, the current EASL-CLIF definition of ACLF had not been established yet. Furthermore, it has to be mentioned that in a subgroup analysis of the Prometheus study, patients with a MELD score >30 showed improved survival (48).

In one recent meta-analysis, which assessed available evidence on ECLS in ACLF of 25 RCTs by the GRADE approach, the authors reported a reduction in mortality (RR 0.84, 95%CI 0.74–0.96) with moderate certainty (50). More recently, a Bayesian network meta-analysis, which included 16 RCTs on artificial and bioartificial support systems in ACLF, concluded that available evidence indicates plasma exchange (PE) in ACLF to be the best treatment option currently available among all support systems (51). In cumulative ranking, PE ranked first and was associated with a significantly increased 3-month overall survival and 3-month transplant-free survival in ACLF patients compared to standard medical treatment. In contrast, other artificial support systems did not reach statistical significance in this meta-analysis (51). Overall, several studies have indicated that PE might be a feasible treatment strategy in ACLF (52, 53). However, due to the low quality of evidence, larger RCTs are required, which are currently undertaken, for example by the APACHE trial.

Taken together, currently available evidence does not support a general recommendation for the use of extracorporeal liver support systems in ACLF patients outside of clinical trials, although under specific circumstances, it could be considered as an option to bridge-to-transplantation (20, 54).

### Transplant Allocation in ACLF

LT is a potentially life-saving treatment for patients with ACLF. This is underscored by the fact that current principles in management of this severe syndrome rely on identification and treatment of the precipitant and supportive care for specific organ failures. Given the high short-term mortality among ACLF patients and in light of the unavailability of specific disease-modifying drugs as well as negative studies regarding albumin dialysis, rescue transplantation emerges as a critical and life-saving option for severe ACLF patients. Data of recent years have accelerated the formation of consensus among societies that patients with ACLF grade 1 and 2 should be listed for LT. In fact, ACLF patients benefit from rapid evaluation and listing, which is highlighted by the observation that even patients who recover from the index ACLF event are still at risk of a recurrent decompensation and more severe ACLF in the future (55). Even after recovery from ACLF, inherent 6-month mortality ranges from 40% to 50% (15, 56).

Patients with AD listed for LT show a waitlist mortality of 15% (57). However, the PREDICT study demonstrated that UDC and pre-ACLF are associated with a significantly higher short-term mortality compared to stable AD, which is not necessarily reflected in current prognostic models. These patients are at risk of developing organ failures and progression to ACLF, resulting in rapid deterioration of their clinical condition (6). Upon progression to ACLF, patients show a 28-day transplant-free mortality ranging from 30 to 40% (10, 15). In patients with ACLF grade 3, 28-day mortality increases to 68%, whereas patients with 4-6 organ failures show an even higher mortality rate of up to 88.9% in 28 days, according to data from the CANONIC study (5). In line with these findings, data analyzed by the United Network for Organ Sharing (UNOS) database showed that patients with ACLF grade 3 had a significantly higher waitlist mortality within 14 days after listing than listed patients with 1a-status (58).

Importantly, this high short-term mortality in severe ACLF patients is not fully reflected by current scoring tools used for transplant allocation. Conventional prognostic models for assessment of mortality risk in patients with cirrhosis are the Model for End-Stage Liver Disease (MELD), MELD-sodium (MELD-Na) and Child-Pugh scores (59, 60). In fact, these scores predict both, progression to ACLF and survival among ACLF patients (61). Most countries have adopted MELD or MELD-Na score-based allocation policies to prioritize most severe patients with decompensated liver cirrhosis for LT. However, these scores lack important clinical determinants of short-term mortality among ACLF patients. For one, no surrogates for SI, such as white blood cell count, CRP or ferritin, are taken into account, although SI is considered the main driver in ACLF progression and strongly correlated with mortality rates (8, 24, 27, 62). Furthermore, neither score incorporates surrogates for portal hypertension or presence of respiratory or circulatory failure to estimate patients’ mortality risk, although recent data suggests that pulmonary failure in particular is an important determinate of mortality in ACLF patients [own unpublished observation]. Also, neither MELD nor MELD-Na score are incorporating cerebral dysfunction/HE. However, the assessment of this clinical parameter could be considered compromised by the subjective nature of its assessment.

In view of these limitations, the CANONIC study specifically designed the CLIF-C ACLF score to assess mortality risk in patients with ACLF (18). The CLIF-C ACLF score incorporates the number of organ failures, reflected by the CLIF-OF score, age and white blood cell (WBC) count as a surrogate for severity of SI (18). These parameters have been determined as crucial predictors of short- and long-term survival in ACLF patients but are not included in other models. In recent years, several studies have corroborated that the CLIF-C ACLF score shows a significantly higher predictive accuracy than other prognostic models for short-term mortality in ACLF patients (18, 63, 64). A CLIF-C ACLF score from 64 to 70 points is regarded as the threshold to futility of care and may thereby be helpful to identify patients in whom supportive care must be critically discussed if rescue LT is not a valid option.

In fact, limitations regarding current MELD score-based risk stratification allocation policies are underlined by a recent study analyzing data from the UNOS database, which showed that patients with ACLF grade 3 and MELD-Na score <25 have a higher waitlist mortality than patients without ACLF and a MELD-Na score >35 (65).

Importantly, a recent study assessed mortality rates in 18,979 patients with ACLF and demonstrated that the MELD-Na score markedly underestimates the 90-day mortality of ACLF patients (66). Moreover, several studies reported a declining predictive accuracy of the MELD score over the last decades of MELD score-based LT allocation. Initially, this became apparent when comparing predictive performance of the MELD score in former studies with reports from current patient populations (16, 18, 60, 67). This observation was corroborated in a recent analysis of 120,156 patients listed for LT between 2002 and 2016 with data provided by UNOS network, displaying a declining MELD score c-statistic of 0.8 in 2002 and only 0.71 in 2016 (68). Multiple reasons for this observation have been proposed, whereby epidemiological shifts in the landscape of cirrhosis with changing prevalence of etiologies and accelerated listing of more highly advanced patients with liver cirrhosis are considered to be major contributors (57). Data suggests that high mortality rates of an increasing number of listed patients with rapid decompensation and ACLF might not be adequately reflected with current prognostic tools. These considerations emphasize the increasing need to improve MELD score-based models to better reflect waitlist mortality and possibly modify and improve LT allocation policies for ACLF patients in the future.

### Patient Selection and Contraindications for LT

**FIGURE 4:**
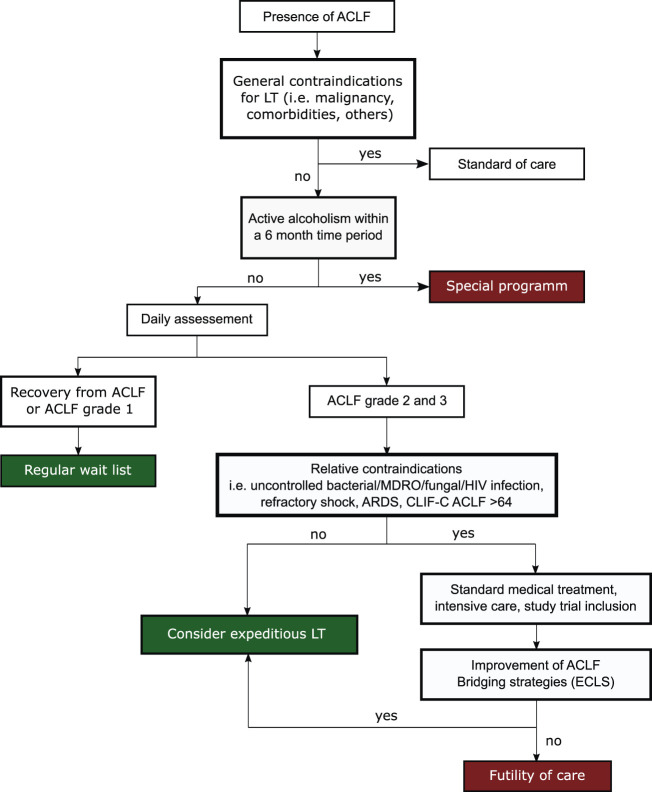
Decision algorithm to assess eligibility of ACLF patients for LT.

In light of these challenges and due to the limited supply of organ donors, optimal patient selection, identification of relative contraindications and timing of LT appears to be critical. A decision algorithm for LT evaluation in ACLF patients is shown in Figure 4.

Recently published data from a large multicentric study identified four independent pretransplant risk factors among patients who received LT in ACLF grade 3 (69). The authors were able to use these risk factors, namely age ≥53 years, lactate level ≥4 mmol/L, mechanical ventilation with pulmonary failure (PaO2/FiO2 ≤ 200 mmHg) and leukocytes ≤10G/L, to develop and validate a prognostic model to predict posttransplant survival in ACLF grade 3 patients. The transplantation for ACLF-3 model (TAM) score assigns 1 scoring point for each criterion met and allows for stratification into two groups: recipients with a TAM score of >2 showed a poor post-LT outcome with a 1-year mortality of almost 84%, while a TAM score of ≤2 was associated with a mortality rate <10% (69).

Although this study provided a novel clinical tool to assess a window of transplantability, one of its major limitations was the fact that the derivation cohort for the TAM score consisted of only 22 patients, who met the inclusion criteria of death within 1 year. A recent single-center study has since retrospectively assessed the TAM score in 100 patients (70). The authors found that the TAM score was efficiently discriminating between ACLF grade 3 post-LT survivors and non-survivors if assessing patients at the time of LT or directly before LT. In contrast, the score did not show any reliable prediction for patient outcome at ICU admission or 2 days after admission (70). Interestingly, a recent study observed that ACLF grade 3 patients, who showed improvement of ACLF severity prior to LT also showed higher post-LT survival rates (71).

The recently published ECLIS study assessed 234 patients receiving LT for ACLF, also reporting pre-LT lactate levels to be predictive of post-LT outcome (72). Furthermore, this study found renal replacement therapy at LT and recent MDRO infection to be independent predictors of poor post-LT outcomes (72). Independent of MDRO status, uncontrolled bacterial infections, fungal infections and severe sepsis are generally considered a contraindication to LT, since post-LT immunosuppression may exacerbate the infection. For a similar reason, uncontrolled human immunodeficiency virus (HIV) infections should also be regarded as relative contraindications to LT (56). However, bacterial infections are the most common ACLF precipitant and, furthermore, are frequent complications upon ACLF progression.

Active alcoholism is considered a contraindication to LT and abstinence for 6 months is a requirement for LT listing in many countries. The 6 month rule is implemented to allow liver recovery, decrease post-LT relapse rates and reduce allograft loss. However, severe alcoholic hepatitis constitutes a major precipitant to ACLF. Due to the high short-term mortality of ACLF, a considerable fraction of patients die within this 6 month period, disregarding whether these patients would otherwise be feasible candidates for LT. This is a controversial topic, since several studies have found that outcomes in LT recipients with alcoholic hepatitis, who received LT in under 6 months showed a high 1-year post-LT survival of 74–94% and relapse rates of 10–17% (73). Evidence is indicating that selective use of LT in patients with alcoholic hepatitis, who meet specific psychosocial requirements, might be a feasible strategy (74, 75). A recent study has introduced the prognostic Sustained Alcohol Use Post-LT (SALT) score, which can be used to identify patients with a low risk of alcohol relapse. The score is comprised of four pre-transplant variables: patients drinking pattern at presentation (>10 drinks/day, +4 points), prior failed rehabilitation attempts (≥2, +4 points), history of alcohol-related legal issues (+2 points) and history of prior non-THC substance abuse (+1 points) (76). In the study cohort, a SALT score of <5 had a 95% negative predictive value and high sensitivity for sustained alcohol use after LT, showcasing that individual patient assessment and selective LT in suitable candidates could be a new approach in LT allocation in the future (76).

### Outcomes After LT

In recent years, several studies have demonstrated high post-LT survival rates among ACLF patients, although data show some geographical variability due to heterogeneity of study populations. Initially, analysis from the CANONIC population showed a 1-year post-LT survival of 75.3% in a small number of 25 ACLF patients receiving LT, which was lower than in the study’s overall population (15).

In the following years, single center retrospective studies have reported 1-year post-LT survival rates ranging from 70% to 87%, depending on patient population and ACLF severity (77-80). A retrospective study conducted by Levesque et al. demonstrated that ACLF patients presenting ACLF grade 1 and 2, according to EASL-CLIF criteria, showed a high 90-day post-LT survival of 85.3% and 83.3%, respectively, while patients transplanted with ACLF grade 3 only had a 90-day survival of 60% in the study population (79). In contrast, a larger multicenter European study including 250 ACLF patients found 1-year post-LT survival of 83.9% in patients with ACLF grade 3 (80), presumably because ACLF grade 3 patients were carefully selected for LT in this study. Acute respiratory distress syndrome (ARDS), uncontrolled sepsis, active gastrointestinal bleeding and hemodynamic instability were considered contraindications to LT in these patients (80). This underlines the importance of patient selection, but urges us not to regard ACLF grade 3 as an absolute contraindication for LT. Moreover, this study strikingly contrasted the 1-year post-LT survival of 83.9% in patients with ACLF grade 3 compared to only 7.9% in the non-LT control group with ACLF grade 3, underlining that LT often is the only life-saving option for patients with severe ACLF.

A recent extensive retrospective analysis has since clearly provided robust data, showing that all ACLF patients, including ACLF grade 3, significantly benefit from LT. Sundaram et al. analyzed data from over 50,000 patients included in the UNOS database and found even higher 1-year survival rates post-LT in ACLF grade 1 (89.1%), ACLF grade 2 (88.1%) and ACLF grade 3 (81.9%) (65). Interestingly, this study found that mechanical ventilation at LT and a donor risk index >1.7 were independently associated with poorer post-LT survival. The donor risk index (DRI) was established to quantitatively assess donor-specific factors to predict the risk of graft failure and is comprised of seven donor characteristics, most importantly donor age, donation after cardiac death or split/partial graft (81).

A more recent study assessing a European cohort of 2,677 patients showed similar results regarding survival, with survival rates being >80% among all ACLF grades (72). Similar results were also found in other prospective and retrospective studies of European cohorts (15, 78). Depending on the study population, risk factors for post-LT mortality in ACLF were mechanical ventilation, circulatory failure and four or more organ failures (65), need for renal replacement therapy, as well as infection with MDROs as precipitating events or as complications (72).

In summary, these studies demonstrate that ACLF patients strongly benefit from LT, and that post-LT survival does not significantly differ from that in patients without ACLF. Furthermore, data urge us to not generally regard ACLF grade 3 as an absolute contraindication for LT, instead patients must be carefully selected.

## Conclusion

In conclusion, various recent studies have demonstrated that LT is a feasible and life-saving option for ACLF patients with excellent post-LT outcomes. In many cases, patients with severe ACLF have no other treatment option than expeditious LT and a clear survival benefit can be shown if patients are carefully selected. Importantly, increased mortality rates among ACLF patients are not fully reflected in current prognostic tools used for transplant allocation. Further studies will be necessary, but data demand a critical reflection of current transplant allocation systems to improve risk stratification in patients with this severe syndrome. In clinical management of decompensated cirrhosis, patient progression to ACLF should trigger early decision-making and rapid transplant evaluation, as suggested for patients with acute liver failure, to stay within the narrow window for transplantation.

## Glossary

ACLF: acute-on-chronic liver failure
AD: acute decompensation
ARDS: acute respiratory distress syndrome
ASH: alcoholic steatohepatitis
ATP: adenosine triphosphate
cACLD: compensated advanced chronic liver disease
CANONIC study: EASL-CLIF Acute oN chrONIC liver failure study
CI: confidence interval
CLIF consortium: chronic liver failure consortium
CLIF-C ACLF score: CLIF-Consortium ACLF score
CLIF-C OFs: CLIF-Consortium Organ Failure score
CLIF-SOFA score: CLIF-sequential organ failure assessment score
CRP: C-reactive protein
DRI: donor risk index
EASL: European Association for the Study of the Liver
ECLIS study: ELITA/EF-CLIF collaborative study
ECLS: extracorporeal liver support systems
FFAs: free fatty acids
GRADE approach: Grading of Recommendations Assessment, Development and Evaluation approach
HCC: hepatocellular carcinoma
HE: hepatic encephalopathy
HIV: human immunodeficiency virus
HNA2: irreversibly oxidized albumin
ICU: intensive care unit
IL-1ra: interleukin-1 receptor antagonist
IL-6: interleukin-6
IL-8: interleukin -8
IMC: intermediate care unit
LT: liver transplantation
MELD score: Model for End-Stage Liver Disease score
MELD-Na score: Model for End-Stage Liver Disease–sodium score
MRDOs: multidrug-resistant organisms
PAMPs: pathogen-associated molecular patterns
PE: plasma exchange
PHT: portal hypertension
Pre-ACLF: pre-acute-on-chronic liver failure
RCT: randomized controlled trial
ROS: reactive oxygen species
RR: relative risk
SALT score: Sustained Alcohol Use Post-LT
SDC: stable decompensated cirrhosis
SI: systemic inflammation
TAM score: transplantation for ACLF-3 model
THC: tetrahydrocannabinol
TIPS: transjugular intrahepatic portosystemic shunt
TNF-α: tumor necrosis factor
UDC: unstable decompensated cirrhosis
UNOS: United Network for Organ Sharing
WBC: white blood cell
